# Detection of cognitive deficits years prior to clinical diagnosis across neurological conditions

**DOI:** 10.1093/braincomms/fcaf307

**Published:** 2025-08-21

**Authors:** Xin You Tai, Sofia Toniolo, David J Llewellyn, Cornelia M van Duijn, Masud Husain, Sanjay G Manohar

**Affiliations:** Nuffield Department of Clinical Neuroscience, University of Oxford, Oxford OX3 9DU, UK; Division of Clinical Neurology, John Radcliffe Hospital, Oxford University Hospitals Trust, Oxford OX3 9DU, UK; Nuffield Department of Clinical Neuroscience, University of Oxford, Oxford OX3 9DU, UK; Division of Clinical Neurology, John Radcliffe Hospital, Oxford University Hospitals Trust, Oxford OX3 9DU, UK; College of Medicine and Health, University of Exeter, Exeter EX1 2HZ, UK; Alan Turing Institute, London NW1 2DB, UK; Nuffield Department of Population Health, University of Oxford, Oxford OX3 7LF, UK; Big Data Institute, Li Ka Shing Centre for Health Information and Discovery, Oxford OX3 7LF, UK; Nuffield Department of Clinical Neuroscience, University of Oxford, Oxford OX3 9DU, UK; Division of Clinical Neurology, John Radcliffe Hospital, Oxford University Hospitals Trust, Oxford OX3 9DU, UK; Department of Experimental Psychology, University of Oxford, Oxford OX2 6GG, UK; Nuffield Department of Clinical Neuroscience, University of Oxford, Oxford OX3 9DU, UK; Division of Clinical Neurology, John Radcliffe Hospital, Oxford University Hospitals Trust, Oxford OX3 9DU, UK; Department of Experimental Psychology, University of Oxford, Oxford OX2 6GG, UK

**Keywords:** cognition, epilepsy, stroke, migraine, multiple sclerosis

## Abstract

Understanding the cognitive trajectory of a neurological disease can provide important insight on underlying mechanisms and disease progression. Cognitive impairment is now well established as beginning many years before the diagnosis of Alzheimer's disease, but pre-diagnostic profiles are unclear for other neurological conditions that may be associated with cognitive impairment. We analysed data from the prospective UK Biobank cohort with study baseline assessment performed between 2006 and 2010 and participants followed until 2021. We examined data from 497 252 participants, aged between 38 and 72 years at baseline, with an imaging sub-sample of 42 468 participants. Using time-to-diagnosis and time-from-diagnosis data in relation to time of assessment, we compared a continuous measure of executive function and magnetic resonance imaging brain measures of total grey matter (GM) and hippocampal volume in individuals with ischaemic stroke, focal epilepsy, Parkinson's disease, multiple sclerosis, motor neurone disease (amyotrophic lateral sclerosis) and migraine. Of the 497 252 participants [226 206 (45.5%) men, mean (SD) age, 57.5(8.1) years], 12 755 had ischaemic stroke, 6758 had a diagnosis of focal epilepsy, 3315 had Parkinson's disease, 2315 had multiple sclerosis, 559 had motor neurone disease and 18 254 had migraine either at study baseline or diagnosed during the follow-up period. Apart from motor neurone disease, all conditions had lower *pre-diagnosis* executive function compared to controls (assessment performed median 7.4 years before diagnosis). At a group level, focal epilepsy and multiple sclerosis showed a gradual worsening in executive function up to 15 years prior to diagnosis, while ischaemic stroke was characterised by a modest decline for a few years followed by a substantial reduction at the time of diagnosis. By contrast, participants with migraine showed a mild reduction in pre-diagnosis cognition compared to controls which improved following clinical diagnosis. Pre-diagnosis MRI GM volume was lower than controls for stroke, Parkinson's disease and multiple sclerosis (scans performed median 1.7 years before diagnosis), while other conditions had lower volumes post-diagnosis. These cognitive trajectory models reveal disease-specific temporal patterns at a group level, including a long cognitive prodrome associated with focal epilepsy and multiple sclerosis. The findings may help to prioritise risk management of individual diseases and inform clinical decision-making.

## Teaser

Tai *et al.* describe distinct pre-diagnosis and post-diagnosis cognitive profiles of several neurological conditions with novel insights such as group-level gradual decline up to 15 years prior to diagnosis in focal epilepsy and multiple sclerosis. Understanding disease-specific patterns of decline may provide early diagnostic and intervention opportunities.

## Graphical Abstract

**Graphical Abstract:**
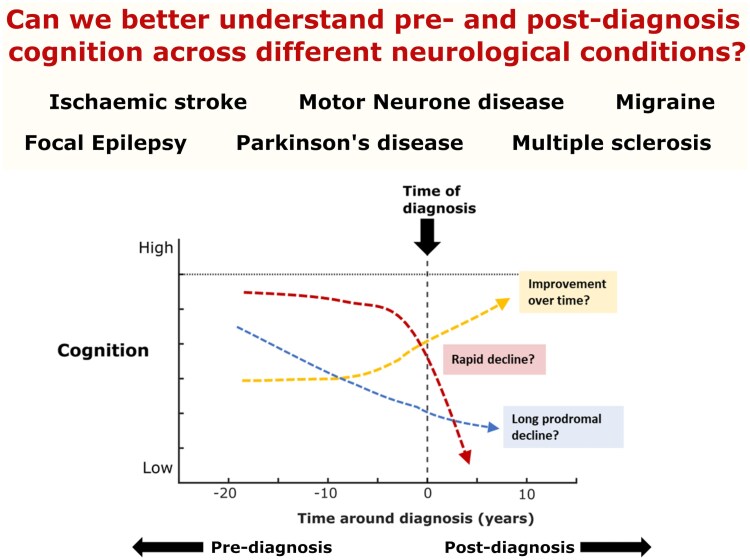

## Introduction

The cognitive profile of a neurological disease across time can provide important insights into underlying mechanisms, response to treatment, disease progression and prognosis. In Alzheimer's disease, cognitive impairment may occur decades prior to the diagnosis^1^ which reflects ongoing accumulation of neurodegenerative protein and associated cellular dysfunction and death.^2^ Following diagnosis, comparison of cognitive trajectories provides an index of response to treatment such as cholinesterase inhibitors^3^ and the recently described amyloid immunotherapies.^4,5^ In other neurological conditions that may cause cognitive impairment, however, pre- and post-diagnosis cognitive profiles are largely unknown.

Longitudinal evaluation is crucial to map cognitive trajectories. Once a diagnosis has been given, individuals with a condition can be followed up with repeat cognitive measurements to understand the post-diagnosis cognitive changes.^6^ Delineating cognition before the disease is diagnosed, however, is less straightforward. The current pre-diagnosis cognitive model of Alzheimer's disease^1^ has been classically derived from autosomal dominant Alzheimer's disease, leveraging families with offspring that have 50% chance of developing the disease.^7^ This cognitive model is corroborated by longitudinal studies of individuals with mild cognitive impairment who progress to develop Alzheimer's disease.^8^

For other neurological conditions, pre-diagnostic assessment is often not feasible, and even if it is, longitudinal evaluation is resource-intensive and logistically difficult. Therefore, many important questions remain unanswered. For example, what is the potential and timeframe of post-stroke cognitive recovery? Many studies describe post-stroke cognitive impairment^9^ but few report data from multiple time points^10^ or performance after 1 year.^11^ Do individuals with focal epilepsy have cognitive impairment prior to starting anti-epileptic medications? One group identified worse memory scores present in new cases of focal epilepsy before treatment initiation,^12^ but precisely when these difficulties begin is unclear. Similarly, what is the cognitive impact of multiple sclerosis and does this improve with treatment? Recent evidence suggests that cortical lesions exist in the early pathogenesis of some people with multiple sclerosis which may affect cognitive function.^13^ However, there is little corroboration outside of small studies. There is some insight into Parkinson's disease with the identification of individuals of rapid-eye movement behavioural disorder (RBD) as a prodromal symptom of the disease. Individuals with RBD have lower scores on neuropsychological testing, including executive function, while individuals with Parkinson's disease and RBD have worse cognitive outcomes compared with those with Parkinson's disease who do not have RBD.^14,15^ In motor neurone disease (amyotrophic lateral sclerosis), there is report of executive function impairment at diagnosis but it is unclear when this begins.^16^ One important related consideration is that cognitive decline may be coupled with reduction in brain volume observed in these conditions.^17-20^

In this study, we use a data-driven approach to understand *both* pre- and post-diagnosis cognitive profiles and neuroimaging data from to the UK Biobank prospective cohort across a range of neurological diseases. Specifically, in relation to date of diagnosis, we model the time-course and magnitude of cognitive impairment across individuals with ischaemic stroke, focal epilepsy, Parkinson's disease, multiple sclerosis, motor neurone disease and migraine, compared to healthy controls. We hypothesized that conditions such as focal epilepsy and Parkinson's disease would be associated with worse cognition many years before the diagnosis is made, whereas individuals with stroke may experience cognitive impairment at the time of diagnosis, as this reflects an index event of brain injury. We also compare the magnitude of cognitive deficit across these neurological conditions to provide a better understanding which may inform clinical decision-making on risk reduction strategies and long-term management.

## Materials and methods

This study examines the UK Biobank, a population-based cohort of over 500 000 participants aged 38–72 years who underwent physiological measurements, cognitive testing and provided biological samples at one of 22 centres across the UK between 2006 and 2010.^21^ A subset re-attended for brain imaging between 2014 and 2020. All participants provided written informed consent. UK Biobank received approval from the North-West Multi-centre Research Ethics Committee (REC number 21/NW/0157).

The primary study objective was to investigate cognition across individuals with ischaemic stroke, focal epilepsy, Parkinson's disease, multiple sclerosis, motor neurone disease and migraine. These diagnoses were chosen as common, acquired neurological conditions that may present to a general neurology clinic. Established cases at study baseline assessment (‘post-diagnosis cases’) as well as new (‘pre-diagnoses’) cases during longitudinal follow-up were identified from hospital inpatient records, coded using the International Classification of Diseases (ICD) ICD-9 and ICD-10 codes, or from death register linkage data as an underlying or contributory cause (UK Biobank codes are found in Supplementary Table 1). Secondary analysis considered all-cause dementia diagnosed after baseline assessment, which provided a benchmark with an expected cognitive decline following diagnosis, and sub-types including Alzheimer's disease, vascular dementia and frontotemporal dementia. For this analysis, we considered on pre-diagnosis data in individuals who were diagnosed following baseline assessment into the study. There were few cases of dementia at baseline assessment (*N*=120), which were excluded due to the direct association with cognitive impairment. Additional exclusion criteria included other neurological conditions such as history of CNS infection, encephalitis, meningitis, haemorrhagic stroke, genetic epilepsies, previous subdural or subarachnoid haemorrhage. Our control group including individuals who did not have one of the neurological conditions of interest, dementia or any of the exclusion criteria.

### Cognitive testing

We analysed data from five computer-based cognitive tasks of working memory or speed of processing as in the previous reports focusing on executive function in the UK Biobank population.^22,23^ The tests were a pairs-matching and snap reaction time, trail-making, tower-rearranging, and symbol-digit substitution tasks, using the first available timepoint. Reaction time was indexed from a variation of the card game, Snap. We calculated accuracy in the Symbol-Digit Substitution task where participants had to match symbol-digit codes to test set of symbols. The number of errors made in the pairs matching task were used, where participants had to memorise the position of six matching card pairs presented simultaneously and identify the location of these pairs after the cards were turned over. The Trail-making task involved linking consecutive numbers (numeric version A) or alphabets and numbers (alphanumeric version B) sequentially. We calculated the difference between completion time for both task versions, an index of executive function.^24^ Accuracy at the Tower Rearranging task, a variation of the Tower of London working memory task, was calculated. In each round, participants were shown three pegs (‘towers’) which had three different coloured rings placed and were asked to indicate the number of moves required to re-arrange the hoops to specific location. The reliability and retest-effects over time for these cognitive tasks have been previously assessed.^25^

### Pre-diagnosis and post-diagnosis cognitive profiles

Group-level cognitive profiles were created in relation to the date of diagnosis whereby post-diagnosis cognition represented all the participants with a condition who were diagnosed prior to baseline study assessment for that individual. For example, the cognitive performance of a participant who was diagnosed with multiple sclerosis 20 years prior to entering the UK Biobank study represented a 20-year post-diagnosis cognitive score for that condition. By contrast, two-year pre-diagnosis cognition reflected cognitive scores at baseline assessment for participants who would (subsequently) be diagnosed with the condition 2 years later during the study follow-up period. Importantly, this is not longitudinal assessment of cognition at an individual level, however, it provides a representation of pre- and post-diagnosis cognitive profiles across time at a group level.

### Main covariates

All full models were adjusted for important confounders including age (continuous), sex (female versus male), education [categorised as higher (college or university degree or other professional qualification), upper secondary (second or final stage of secondary education), lower secondary (first stage of secondary education), vocational (work-related qualifications), or other], socioeconomic status (categories derived from Townsend deprivation index^26^ quintiles 1, 2 to 4 and 5).

### Brain imaging variables

Magnetic resonance imaging (MRI) data were acquired on a Skyra 3T scanner (Siemens; Munich, Germany) including high-resolution, T1-weighted, three-dimensional magnetisation-prepared gradient echo structural images and T2-weighted fluid-attenuated inversion recovery images. Full imaging protocols and processing pipeline have been previously described.^27^ We examined a global brain measure of total grey matter (GM) volume, useful to assess wide-spread change, and the total hippocampal volume which is related to cognition and relevant for several of the diseases such as epilepsy^28^ and stroke.^29^ Median absolute deviation was used to exclude outliers, and volumes were adjusted for potentially confounding baseline measures of age, age squared, head size, and imaging site.^27^

### Statistical analysis

Confirmatory factor analysis was performed on cognitive variables to produce a continuous, summary latent measure of working memory and reaction time which we termed ‘Executive Function’ for simplicity (this method has been previously described in detail).^23,30^ Estimating a latent variable has the methodological advantage of controlling measurement error that can artificially reduce the relationship between measured variables in standard univariate analyses.^31^ Missing cognitive data were estimated using full information maximum likelihood, which gives unbiased parameter estimates and standard errors.

The relationship between Executive Function across age was first examined for each group—ischaemic stroke, focal epilepsy, Parkinson's disease, multiple sclerosis, motor neurone disease and migraine. Previous work has identified age as the most important confounder when assessing cognition.^30^ We therefore controlled for age in two different ways for robustness: using a residual-based, sliding window approach with fixed age-quantile widths moved along the age distribution (using a 20% gaussian kernel,^23,32^ code: conditionalPlot.m available: https://osf.io/vmabg/). This model-free approach has the methodological advantage of representing nonlinearities in the data and is not constrained by a pre-specified model. The second method to account for age as a confound was a more traditional approach by including age as a covariate in a general linear model together with other important baseline characteristics including sex, education level and socioeconomic status. The difference in Executive Function between pre-diagnosis cases, post-diagnosis cases and the control group was compared using ANOVA with post-hoc Tukey analysis to account for pairwise or multiple comparisons, *P*-values were two-sided with statistical significance set at *P* < 0.05 for all analyses. Brain measures were analysed using the same methods.

The age-residualised cognitive scores and brain measures were visualised for each group using the same sliding window, model-free approach with fixed quantile widths of time in relation to diagnosis. We compared this data with age-residualised cognitive scores from the control group. Motor neurone disease was not included in this visualisation due to low sample size but was analysed when comparing mean pre- and post-diagnosis Executive Function and brain imaging volumes. For the main exposures and covariates, there were less than 3% missing or not known data and complete case analysis was applied. New diagnoses were included if recorded between baseline until the date of first diagnosis, death, loss to follow-up, or the last surveyed hospital admission date (31 March 2021, for England and Scotland and 28 February 2018, for Wales), whichever came first. These censoring dates were recommended by UK Biobank as the data was estimated to be over 90% complete in England, Scotland and Wales. Analyses were done in Matlab R2018a or in R version 4.0.3 using the Lavaan^33^ or survival package.

## Results

The UK Biobank cohort comprised 502 536 participants at baseline. After excluding those who did not meet the inclusion criteria (*N* = 7216), our study included 497 252 individuals (flowchart in Supplementary Fig. 1). Overall, participants had a mean (SD) age of 57.5 (8.1) years and 54.5% were female. At study baseline, there were 6352 with ischaemic stroke, 4247 participants with epilepsy, 873 with Parkinson's disease, 1861 with multiple sclerosis, 70 participants diagnosed with motor neurone disease and 14 588 with migraine. The median time-since-diagnosis to baseline study assessment in these post-diagnosis cases ranged from 27.6 years in individuals with migraine to 3.3 years in individuals with motor neurone disease.

Over a follow-up period of 12.0 median years (interquartile range 11.2–12.7 years and 5 826 307 total follow-up years), there were 6403 participants with a new diagnosis of ischaemic stroke, 2511 participants with focal epilepsy, 2442 with Parkinson's disease, 454 with multiple sclerosis, 489 with motor neurone disease and 3666 with migraine. The median time-to-diagnosis was 7.4 years from baseline study assessment in these ‘pre-diagnosis’ cases across all conditions. Individual condition demographic information can be found in Supplementary Table 2 while histograms of the diagnosis dates in relation to baseline study assessment are shown in Supplementary Fig. 2.

### Pre-diagnosis and post-diagnosis executive function across neurological conditions

**Figure 1:**
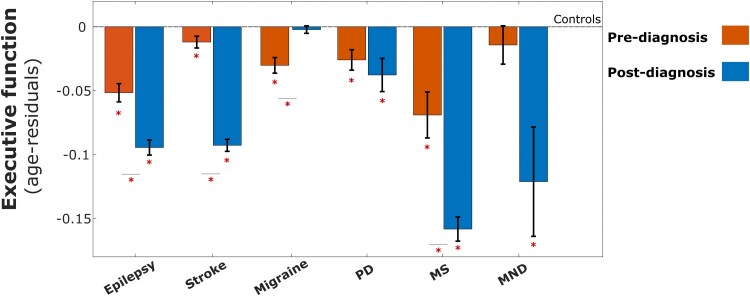
**Pre-diagnosis and post-diagnosis Executive Function across different neurological conditions.** Prior to being diagnosed, participants with epilepsy, stroke, migraine, Parkinson's disease and multiple sclerosis have a lower Executive Function (*z*-scored values) compared to controls, with a median time-to-diagnosis of 7.4 years. Dotted baseline represents level of Executive Function in controls. Across all neurological conditions, apart from migraine and motor neurone disease, Executive function was lower in individuals with an established diagnosis (post-diagnosis cognition) compared to participants who would be diagnosed during the follow-up period of the study. Participants with migraine had a higher post-diagnosis Executive Function compared to pre-diagnosis participants. Error bars denote standard error. Mean executive function for each condition was compared with control group, asterisk (*) below each bar denotes significant difference *P* < 0.05, while within-condition pre-diagnosis versus post-diagnosis executive function was compared and represented by an asterisk across condition bars using a post-hoc Tukey analysis to account for multiple comparison. For focal epilepsy, stroke, Parkinson's disease, migraine, multiple sclerosis, and motor neurone disease, the individual pre-diagnosis statistics compared with controls were: *N* = 2439, *t* = −5.89, *P* < 0.001; *N* = 6288, *t* = −2.52, *P* = 0.01; *N* = 3611, *t* = −3.46, *P* < 0.001; *N* = 2384, *t* = −2.99, *P* < 0.001; *N* = 452, *t* = −3.98, *P* < 0.001 and *N* = 485, *t* = −0.20, *P* = 0.84, respectively, while the post-diagnosis statistics compared with controls were: *N* = 4159, *t* = −12.87, *P* < 0.001; *N* = 6201, *t* = −17.00, *P* < 0.001; *N* = 14480, *t* = −4.43, *P* < 0.001; *N* = 856, *t* = 0.64, *P* = 0.52; *N* = 1,825, *t* = −17.33, *P* < 0.001 and *N* = 70, *t* = −1.98, *P* = 0.05, respectively. PD, Parkinson's disease; MS, multiple sclerosis; MND, motor neurone disease.

A continuous cognitive function latent variable of Executive Function was estimated from five cognitive tasks of working memory or speed of processing (model and fit indices shown in Supplementary Fig. 3). Age is often the most important confounder when considering cognition and, consistent with previous findings,^23,30^ Executive Function declined uniformly with age among all groups (Supplementary Fig. 4). We therefore controlled for age using a residual-based method. Executive Function scores were on average lower in individuals with an established diagnosis (post-diagnosis) compared to individuals before they were diagnosed with a neurological condition (pre-diagnosis), apart from in those with migraine and motor neurone disease (Fig. 1, Supplementary Fig. 5 shows individual condition plots and Supplementary Table 3 with post-hoc Tukey analysis results, controlling for multiple comparison testing). Further, pre-diagnosis Executive Function scores were significantly lower than controls for all conditions apart from motor neurone disease. For robustness, we used a separate general linear model to corroborate these findings and control for other covariates including sex, level of education and socioeconomic status. Similar patterns of differences were observed when comparing pre-diagnosis and post-diagnosis Executive Function scores with controls (Supplementary Table 4).

### Modelling cognitive trajectories across time in relation to diagnosis

**Figure 2:**
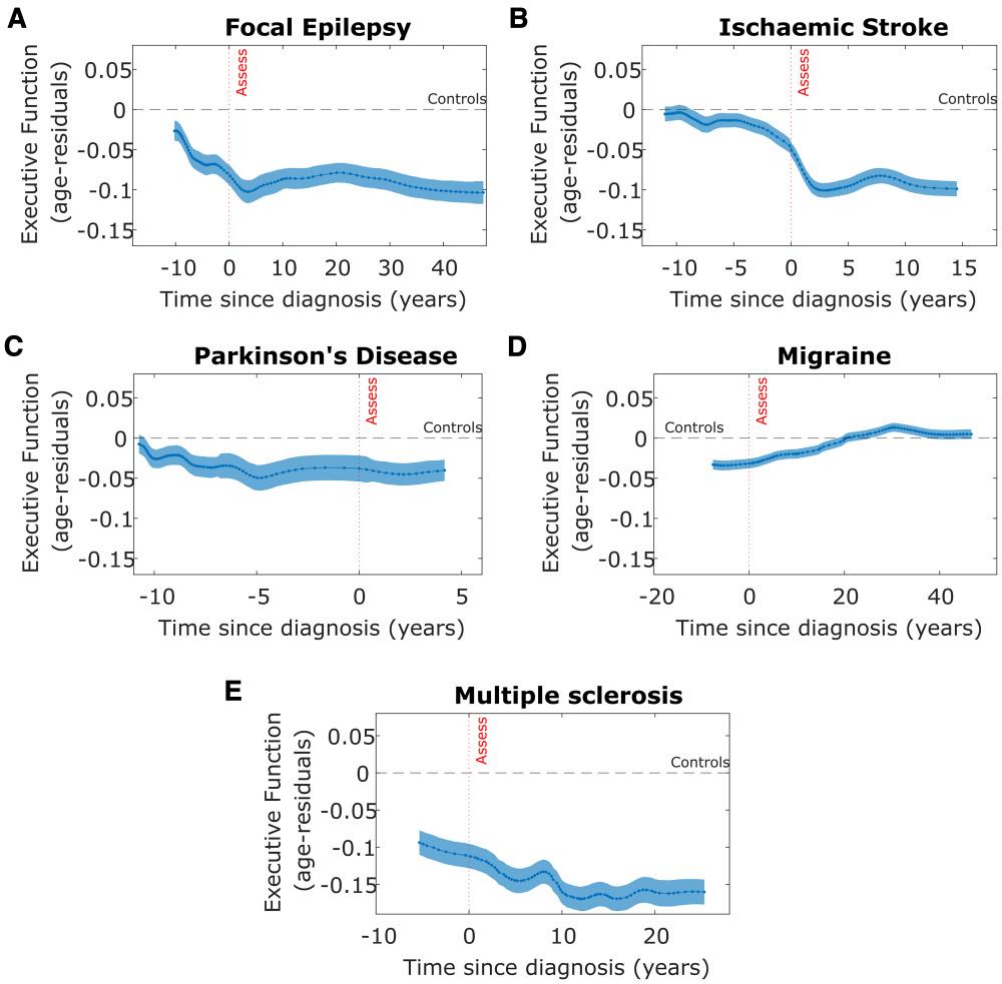
**Cognitive profile across time in relation to the diagnosis of disease across different neurological conditions.** Executive Function (age-residuals, *z*-scored values) in relation to time of diagnosis across participants with different neurological conditions represents a pre- and post-diagnosis cognitive trajectory. Dashed line indicates the level of Executive Function in controls. Dense dotted line indicates the study assessment time. Negative time values reflect individuals who *will develop* the disease (pre-diagnosis cases) during the study follow-up period. Participants with **A** focal epilepsy (*N* = 6758), **C** Parkinson's disease (*N* = 3315) and **E** multiple sclerosis (*N* = 2315) show declining cognitive scores prior to diagnosis at a group level. Cognitive scores plateau after diagnosis in several conditions, including focal epilepsy, and further declines in multiple sclerosis. Parkinson's disease showed low cognitive scores throughout. **B** Stroke (*N* = 12 755) was associated in a sharp decline in Executive Function at the time of diagnosis while participants in **D** migraine (*N* = 18 254) improved following diagnosis to a level similar to controls. *Assess—time of study assessment*.

Using a nonlinear approach, Fig. 2 shows the age-residual Executive Function scores leading up to and after diagnosis for each neurological condition (Fig. 2). The upper limit of this time-course reflects participants who were diagnosed many years before entering the UK Biobank study and this was different for each neurological condition. The lower limit of these cognitive trajectories represents individuals who were diagnosed during the follow-up period of the UK Biobank study (up to 15 years follow-up after baseline assessment).

This approach identified disease-specific patterns in cognitive profiles at the group level. Executive Function gradually declined across the focal epilepsy group over 10 years before a diagnosis was made whereas participants with stroke showed Executive Function scores similar to controls up until a few years before their event, then a modest worsening followed by a substantial decline at diagnosis, likely consistent with their index event. The multiple sclerosis group had comparatively worse Executive Function compared to all other neurological conditions assessed, with a gradual decline pre-diagnosis which continued post-diagnosis. By contrast, the migraine group had lower Executive Function before their diagnosis which improved to the baseline control level after the diagnosis was made. Following diagnosis, there was an initial plateau in Executive Function was observed in the epilepsy and stroke groups with a small improvement between 2 and 10 years observed in stroke but not to the level of controls. Individuals with Parkinson's disease showed a constant lower-than-control level of Executive Function 10 years before their diagnosis. For motor neurone disease, there was mainly data available leading up to diagnosis with few established cases at study baseline. There was worse post-diagnosis Executive Function in the motor neurone disease group although this was associated with large standard error due to smaller sample size.

### Pre-diagnosis cognitive profile in dementia and sub-types

There were 5963 participants who developed all-cause dementia during the follow-up period of the study. Executive Function declined progressively in individuals who were closer to receiving the diagnosis (Supplementary Fig. 6). This pattern was similar when considering those who were diagnosed with Alzheimer's disease (2420 participants), vascular dementia (1019 participants) and other dementias (2325 participants) but was not observed in participants with frontotemporal dementia (199 participants).

#### Total GM pre-diagnosis and post-diagnosis across neurological conditions

**Figure 3:**
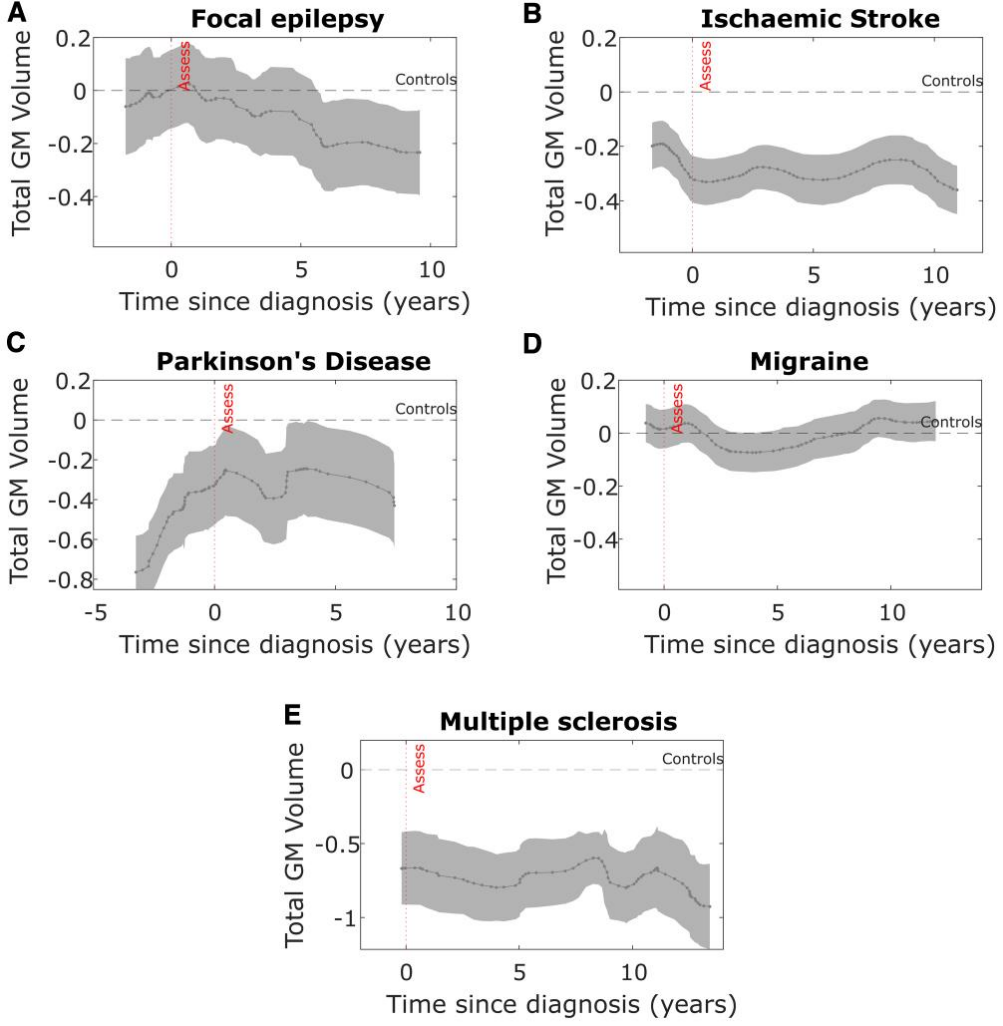
**Total GM volume across time in relation to diagnosis across different neurological conditions.** Total GM volume (age-residuals, *z*-scored) in relation to time of diagnosis across different neurological conditions. Dashed line indicates the total GM volume in controls. Densely dotted line indicates time of the brain scan event. Negative time values reflect individuals who will develop the disease (pre-diagnosis cases) after the brain scan has been performed. At a group level, in participants with **A** epilepsy (*N* = 370), post-diagnosis total GM volume decreases more steeply than expected for age in participants who have had the disease longer. Total GM volume in participants with **E** multiple sclerosis (*N* = 170), **C** Parkinson's disease (*N* = 94) and a history of **B** ischaemic stroke (*N* = 636) are lower than controls before and after the diagnosis has been made. Participants with **D** migraine (*N* = 1973) have a similar age-corrected total GM volume throughout the time course. *Assess—time of study assessment*.

A subset of the UK Biobank cohort underwent MR brain imaging (*N* = 42 468) following baseline study assessment (range 3.8 to 13.8 years after, median 9.2 years). Since imaging was obtained later, the median time-to-diagnosis from imaging acquisition was lower at 1.7 years than cognitive assessment (Supplementary Table 5 details the breakdown for each neurological condition). We used the same approach in modelling the pre- and post-diagnosis time-course for total GM, adjusted for age, across each neurological condition (time-course limited to 20 years post-diagnosis for visualisation in Fig. 3). Total GM volume was lower than controls in individuals who have had an ischaemic stroke and those with multiple sclerosis and Parkinson's disease, both pre- and post-diagnosis (general linear model showing statistical differences in Supplementary Table 6). The epilepsy group showed decreasing total GM volume post-diagnosis but have volumes similar to controls leading up to the diagnosis. Total GM volume in participants in migraine are comparable to controls before and after the diagnosis. The results for participants with motor neurone disease were not visualised because only 19 participants had imaging data.

The same pre- and post-diagnosis imaging analysis was performed for total hippocampal volume which showed lower pre- and post-diagnosis volume in participants with multiple sclerosis only, while ischaemic stroke and focal epilepsy were associated with lower post-diagnosis hippocampal volume only (Supplementary Table 7), suggesting that different disorders may be weighted towards affecting different brain areas.

## Discussion

This study examined pre- and post-diagnosis cognitive profiles in large groups of individuals with ischaemic stroke, focal epilepsy, migraine, Parkinson's disease, multiple sclerosis and motor neurone disease. By leveraging cross-sectional cognitive data at baseline study assessment along with time-to-diagnosis (in the future) and time-since-diagnosis (from the past), we analysed group-level cognitive profiles up to 15 years pre-diagnosis and over 40 years prior to the diagnosis. These profiles offer insight into cognitive ‘trajectories’ at a group level which have not been clearly described previously for several of these conditions. Our residual-based, sliding-window analysis allows nonlinearities to emerge from the data which more closely approximates real-world disease characteristics rather than constraining analysis to linear or quadratic functions. All condition groups, apart from motor neurone disease, showed a measurable cognitive deficit several years before diagnosis compared to controls, with multiple sclerosis having the lowest cognitive score across all conditions.

Our findings offer several potential disease-specific insights. Focal epilepsy has been closely linked to cognitive impairment,^34^ however, this has been difficult to dissociate from cognitive side-effects of anti-seizure medication.^12^ In the present study, impaired cognition was evident over a decade prior to the diagnosis. This may reflect ongoing sub-clinical epileptic activity^35^ or associated neuropathology^36^ before two overt seizures occur which are required for diagnosis, or a seizure with pathology on imaging or electroencephalography.^37^ Cognitive scores plateaued across the group within 2 years following focal epilepsy diagnosis which may be due to treatment stability before further decline after 20 years. Pre-diagnosis cognitive deficits observed with multiple sclerosis may reflect emerging research on early B-cell related, cortical pathology that can occur before the characteristic white matter demyelinating lesions.^13,38^

A sharp decline in cognition was observed around the diagnosis of ischaemic stroke, likely reflecting the index stroke event, while a modest reduction in cognitive scores in the few years prior may indicate underlying small vessel disease related to vascular risk factors.^30,39^ This supports that minor cognitive difficulties should prompt a review of vascular risk factors in the older general population. Interestingly, a small increase in cognitive scores between up to 8 years following stroke diagnosis was observed which warrants further analysis and may relate to factors of stroke rehabilitation and brain recovery processes such as neuroplasticity.^40,41^ By contrast, the migraine group had lower cognitive scores leading up to the diagnosis that improved post-diagnosis back to a level similar to controls. This may represent generalised cognitive dysfunction of attention and working memory seen with migraine,^42^ commonly described as a ‘brain fog’, but with a potentially beneficial treatment effect following diagnosis.^43^ Importantly, the lack of ongoing cognitive decline is in line with the absence of underlying progressive or degenerative pathology in migraine.^44^ Individuals with Parkinson's disease had low cognitive scores both pre- and post-diagnosis. This may reflect the underlying alpha-synucleinopathy that likely occurs many years prior to the diagnosis and is evidenced by early non-motor symptoms of anosmia and non-severe autonomic dysfunction that clinicians examine for when assessing a potential patient.^45,46^ Our findings identified lower pre-diagnosis cognitive scores in individuals with motor neurone disease which expands on the report of executive dysfunction at diagnosis,^16^ however, there were too few post-diagnosis cases in this cohort to identify potential change. The pre-diagnosis executive function for the motor neurone disease group was similar to controls with a large standard error. This likely reflects the sample size and may also suggest this group comprises primarily with pure motor presentations of motor neurone disease rather than mixed motor and frontal behavioural phenotypes. Similarly, sample size may explain the lack of clear executive function decline in individuals with FTD prior to diagnosis. Furthermore, individuals who may manifest significant behavioural changes and executive dysfunction as part of the FTD spectrum may have been less inclined to participate in the study.

The neuroimaging findings suggest that reduced global brain volume can be detected in participants with ischaemic stroke, Parkinson's disease and multiple sclerosis several years before the diagnosis. While this is consistent with the neurodegenerative and neuroinflammatory processes in Parkinson's disease^47^ and multiple sclerosis,^38^ respectively, this has not been clearly reported before. This supports older literature in multiple sclerosis showing biochemical changes in both white matter lesions and normal looking white matter.^48^ These findings suggest that this approach may offer insight into pre-diagnostic brain structure and will benefit from a closer examination of different brain regions which may be specifically involved in each condition.

Several further observations can be made for each individual condition, but it is important to consider our findings in the context of the study limitations. Firstly, this is a broad overview into each condition as disease-specific characteristics have not been accounted for, such the location of demyelinating lesions in participants with multiple sclerosis or sub-type of focal epilepsy. This information was not available from this population cohort. Medications may be more important for some of the studied diseases as, for example, we have previously shown that the number of anti-seizure medications correlates to worse cognition likely as a marker of seizure severity rather than just a medication side effect.^22^ In-depth analysis in each disease is the subject of ongoing investigation. However, in our view, this general approach provides a useful and novel comparison across these conditions and benefits may include health service planning and economic modelling particularly in the context of uncommon neurological disorders with little to no longitudinal data.

Crucially, due to the observational nature of this cohort, any associations or findings cannot be taken as causal and potential mechanistic explanations are hypothetical. Diagnosis information was based on hospital records or death certificate data and therefore delayed or incorrect diagnoses may affect the cohort selection. This may explain nonetheless, early changes were observed up to 20 years before diagnosis for several of these conditions. Electronic records may be more accurate for certain conditions, such as ischaemic stroke, when there is usually a clear event that brings an individual to hospital compared to neurodegenerative conditions with a longer diagnostic journey. However, the sample size of each sub-group is an order of magnitude larger than most separate studies of each condition and will help identify small effects within the data. It is important to emphasize that this data is not longitudinal at an individual level. The cross-sectional nature of the data means that these cognitive and brain models likely capture a combination of actual disease trajectory over time and cohort-specific characteristics. These characteristics include a selection bias that reflects the relatively healthy nature of the UK Biobank.^49^ However, this healthy effect described may underestimate the magnitude of cognitive changes should a more representative sample of each condition were considered. The geographic location of the data coming from the UK is another point to note and may not be generalisable to other geographic populations. Further in-depth longitudinal studies for each condition would be helpful to corroborate our findings, keeping in mind the cost to perform such studies at scale. This would be facilitated through remote, digital cognitive testing that is becoming more readily available.^50^

## Conclusion

This study of cognitive and brain trajectories has offered novel disease-specific insights and may improve our understanding of underlying pathological mechanisms. At a group level, cognitive deficits were identified over a decade before diagnosis in conditions such as epilepsy and multiple sclerosis while cognition post-diagnosis improved to a healthy control baseline in individuals with migraine but declined across other conditions. This nonlinear approach using cross-sectional cognitive data with time-to-diagnosis and time-since-diagnosis is made possible with the large cohort of the UK Biobank and is a cost-effective and useful method to understand disease time-courses compared to longitudinal studies. These findings may prioritise risk management of individual diseases and may inform screening, clinical diagnosis and long-term decision-making such as prioritising cognitive health from the onset of diagnosis.

## Supplementary Material

fcaf307_Supplementary_Data

## Data Availability

The data analysed during the current study are available from the UK Biobank https://www.ukbiobank.ac.uk/researchers/. The variables used are detailed in Supplementary Table 1. This research was conducted using the UK Biobank resource under application 9462. Code to generate the cognitive trajectory figures using conditionalPlot.m is available: https://osf.io/vmabg/
