# Supplementary material for: Detection of cognitive deficits years prior to clinical diagnosis across neurological conditions
Source: Brain Commun. 2025 Aug 21;7(5):fcaf307. doi: 10.1093/braincomms/fcaf307 (PMC12411756; doi:10.1093/braincomms/fcaf307)
Supplement: fcaf307_Supplementary_Data [file fcaf307_supplementary_data.pdf]

Supplementary material

Supplementary Table 1. Codes used in the UK Biobank study to identify dementia and cardiometabolic condition cases and exclusion diagnoses

Supplementary Table 2. Baseline characteristics across different study sub-groups

Supplementary Table 3. Post-hoc comparisons examining the differences in Executive Function between controls, pre-diagnosis and postdiagnosis scores across different neurological conditions

Supplementary Table 4. Comparing pre-diagnosis and post-diagnosis Executive Function in each neurological condition using a general linear model controlling for other baseline characteristics

Supplementary Table 5. Number of participants in the imaging sub-cohort and temporal relation to diagnosis across different neurological conditions

Supplementary Table 6. Comparing pre-diagnosis and post-diagnosis total grey matter volume in each neurological condition using a general linear model controlling for other baseline characteristics

Supplementary Table 7. Comparing pre-diagnosis and post-diagnosis total hippocampal volume in each neurological condition using a general linear model controlling for other baseline characteristics

Supplementary Figure 1. Study flowchart

Supplementary Figure 2. Histogram of when neurological conditions were diagnosed in relation to study assessment.

Supplementary Figure 3. Confirmatory factor analysis of computer-based cognitive tasks

Supplementary Figure 4. Executive Function across age for different neurological conditions

Supplementary Figure 5. Comparing pre-diagnosis and post-diagnosis Executive Function for each individual neurological condition

Supplementary Figure 6. Pre-diagnosis cognitive profile for participants with dementia

Supplementary Table 1. Codes used in the UK Biobank study to identify dementia and cardiometabolic condition cases and exclusion diagnoses

	**Algorithmically-derived**	**Self-report (includes non-cancer and treatment self report)**	**Illness code: ICD-10**	**Illness code: ICD-9**
**Dementia**	-	1263	AD: F00, F00.0, F00.1, F00.2, F00.9, G30, G30.0, G30.1, G30.8, G30.9 VaD: F01, F01.0, F01.1, F01.2, F01.3, F01.8, F01.9, I67.3 FTD: F02.0, G31.0, Other codes for all-cause dementia: A81.0, F02, F02.1, F02.2, F02.3, F02.4, F02.8, F03, F05.1, F10.6, G31.1, G31.8	AD: 331.0 VaD: 290.4 FTD: 331.1 Other codes for all-cause dementia: 290.2, 290.3, 291.2, 294.1, 331.2, 331.5
**Stroke**	All-cause stroke: 42006	1081	I630, I631, I632, I633, I634, I635, I636, I638, I639,	43491
**Epilepsy**	-	1264	G40, G400, G401, G402, G405, G406, G407, G408, G409, G41, G410, G411, G412, G418,G419	34540, 34540, 34541, 34550, 34551
**Migraine**		1265	G43, G430, G431, G432, G433, G438, G439	34690
**Motor Neurone Disease (Amyotrophic lateral sclerosis)**	-	1259	G122	3352
**Multiple Sclerosis**	-	1261	G35, G350	-
**Infection of the central nervous system***	-	1244	-	-
**Encephalitis***	-	1246	-	-
**Meningitis***	-	1247	-	-
**Head Injury***	-	1266	-	-
**Subdural Haematoma***	-	1083	-	-
**Subarachnoid Haemorrhage***	-	1086	-	-

*denotes exclusion condition

Supplementary Table 2. Baseline characteristics across different study sub-groups

Characteristic	Controls N (%)	Epilepsy N (%)	Stroke N (%)	Parkinson's Disease N (%)	Migraine N (%)	Multiple sclerosis N (%)	Motor Neurone disease N (%)
total N	453470 (91.20)	6758 (1.36)	12755 (2.67)	3315 (0.67)	18254 (3.67)	2315 (0.47)	559 (0.11)
Age, mean (SD), years	57.35 (8.09)	58.15 (8.11)	62.24 (6.66)	63.95 (5.29)	56.32 (7.95)	56.16 (7.66)	61.68 (6.64)
Sex							
Female	245508 (54.14)	3397 (50.27)	5099 (39.98)	1265 (38.16)	14105 (77.27)	1679 (72.53)	245 (43.83)
Established diagnoses at study baseline (post-diagnosis)	-	4247 (0.85)	6352 (1.28)	873 (0.18)	14588 (2.93)	1861 (0.37)	70 (0.01)
Median time since diagnosis (to study baseline), *years*	-	23.74	5.59	4.15	27.61	12.46	3.28
New diagnoses after study baseline (pre-diagnosis)	-	2511 (0.51)	6403 (1.29)	2442 (0.49)	3666 (0.74)	454 (0.09)	489 (0.10)
Median time to diagnosis (after study baseline), *years*	-	-7.21	-7.59	-8.44	-7.62	-6.61	-6.78
Education[a]							
Higher	212761 (46.92)	2540 (37.59)	4543 (35.62)	1377 (41.54)	8876 (48.62)	1129 (48.77)	227 (40.61)
Upper Secondary	57133 (12.6)	858 (12.7)	1517 (11.89)	347 (10.47)	2211 (12.11)	225 (9.72)	61 (10.91)
Lower Secondary	24699 (5.45)	333 (4.93)	580 (4.55)	176 (5.31)	1043 (5.71)	154 (6.65)	22 (3.94)
Vocational	75246 (16.59)	1166 (17.25)	1963 (15.39)	484 (14.6)	3191 (17.48)	438 (18.92)	96 (17.17)
Other	83631 (18.44)	1861 (27.54)	4152 (32.55)	931 (28.08)	2933 (16.07)	369 (15.94)	153 (27.37)
Socioeconomic status quintile[b]							
1 (least deprived)	91440 (20.16)	1041 (15.4)	2025 (15.88)	721 (21.75)	3572 (19.57)	467 (20.17)	113 (20.21)
2-4	272574 (60.11)	3777 (55.89)	7186 (56.34)	1933 (58.31)	10970 (60.1)	1391 (60.09)	334 (59.75)
5 (most deprived)	88891 (19.6)	1938 (28.68)	3529 (27.67)	656 (19.79)	3686 (20.19)	453 (19.57)	110 (19.68)
n/a	565 (0.12)	2 (0.03)	15 (0.12)	5 (0.15)	26 (0.14)	4 (0.17)	2 (0.36)

Percentages may not sum to 100 because of rounding.

[a] Higher education defined as college/university degree or other professional qualification; upper secondary, second/final stage of secondary education; lower secondary, first stage of secondary education; vocational, work-related practical qualifications.

[b] Socioeconomic status assessed on the Townsend deprivation index, which combines information on social class, employment, car availability, and housing.

Supplementary Table 3. Post-hoc comparisons examining the differences in Executive Function between controls, pre-diagnosis and postdiagnosis scores across different neurological conditions

	Epilepsy		Stroke		Parkinson's disease		Migraine		Multiple Sclerosis		Motor Neurone disease	
	**F**	**p**	**F**	**p**	**F**	**p**	**F**	**p**	**F**	**p**	**F**	**p**
**Overall model**	189.70	*< .001	235.08	*< .001	14.31	*< .001	18.48	*< .001	202.40	*< .001	5.05	*< .001
**Post-hoc analysis**	**Mean diff (95% CI)**	$\mathbf{P_{Tukey}}$	**Mean diff (95% CI)**	$\mathbf{P_{Tukey}}$	**Mean diff (95% CI)**	$\mathbf{P_{Tukey}}$	**Mean diff (95% CI)**	$\mathbf{P_{Tukey}}$	**Mean diff (95% CI)**	$\mathbf{P_{Tukey}}$	**Mean diff (95% CI)**	$\mathbf{P_{Tukey}}$
**Individual groups**												
Controls – Pre-diagnosis	0.06 (0.04 - 0.07)	*< .001	0.02 (0.01 - 0.03)	*< .001	0.03 (0.01 - 0.05)	*< .001	0.03 (0.02 - 0.05)	*< .001	0.07 (0.03 - 0.11)	*< .001	0.02 (-0.02 - 0.06)	0.5
Controls – Post-diagnosis	0.1 (0.09 - 0.11)	*< .001	0.1 (0.09 - 0.11)	*< .001	0.04 (0.01 - 0.07)	*< .001	0.01 (0.00 - 0.01)	0.11	0.16 (0.14 - 0.18)	*< .001	0.12 (0.03 - 0.22)	*0.01
Pre-diagnosis – Post-diagnosis	0.04 (0.02 - 0.06)	*< .001	0.08 (0.07 - 0.10)	*< .001	0.01 (-0.02 - 0.04)	0.68	-0.03 (-0.04 – -0.01)	*< .001	0.09 (0.05 - 0.13)	*< .001	0.11 (0 - 0.21)	*0.05

Post-hoc analysis of relationship between individual groups of controls, pre-diagnosis cognition and post-diagnosis cognition for each neurological conditions with a Tukey correction applied to account for multiple comparisons. CI- confidence interval.

Supplementary Table 4. Comparing pre-diagnosis and post-diagnosis Executive Function in each neurological condition using a general linear model controlling for other baseline characteristics

	Epilepsy			Stroke			Parkinson's disease			Migraine			Multiple Sclerosis			Motor Neurone disease		
	**Full Model[a]**			**Full Model[a]**			**Full Model[a]**			**Full Model[a]**			**Full Model[a]**			**Full Model[a]**		
	**β**	**t-stat**	**p**	**β**	**t-stat**	**p**	**β**	**t-stat**	**p**	**β**	**t-stat**	**p**	**β**	**t-stat**	**p**	**β**	**t-stat**	**p**
**Executive function**																		
Controls		Baseline			Baseline			Baseline			Baseline			Baseline			Baseline	
Pre-diagnosis	-0.05	-5.89	*< .001	-0.01	-2.52	0.01	-0.03	-3.46	*< .001	-0.02	-2.99	*< .001	-0.07	-3.98	*< .001	0.00	-0.20	0.84
Post-diagnosis	-0.08	-12.87	*< .001	-0.09	-17.00	*< .001	-0.06	-4.43	*< .001	0.00	0.64	0.52	-0.15	-17.33	*< .001	-0.10	-1.98	0.05
**Age**	-0.01	-171.33	*< .001	-0.01	-171.51	*< .001	-0.01	-170.61	*< .001	-0.01	-173.64	*< .001	-0.01	-170.73	*< .001	-0.01	-170.67	*< .001

[a]*Full model: General linear model adjusted for baseline characteristics including age, sex, education, socioeconomic status and assessment centre.* β = *unstandardised betas*

Supplementary Table 5. Number of participants in the imaging sub-cohort and temporal relation to diagnosis across different neurological conditions

Characteristic	Controls	Epilepsy	Stroke	Parkinson's Disease	Migraine	Multiple sclerosis	Motor Neurone disease
	N (%)	N (%)	N (%)	N (%)	N (%)	N (%)	N (%)
Total N (imaging cohort)	42468 (93.57)	370 (0.87)	636 (1.50)	94 (0.22)	1973 (4.65)	170 (0.40)	19 (0.04)
Established diagnoses at brain scan visit (post-diagnosis)	-	321 (0.76)	468 (1.10)	56 (0.13)	1852 (4.36)	155 (0.36)	3 (0.07)
Median time since diagnosis (to brain scan), years	-	27.36	10.87	6.25	33.68	17.00	2.62
New diagnoses after brain scan visit (pre-diagnosis)	-	49 (0.12)	168 (0.40)	38 (0.09)	121 (0.28)	15 (0.04)	16 (0.04)
Median time to diagnosis (after brain scan), years	-	-1.80	-1.60	-2.26	-1.56	-1.39	-1.97

Percentages may not sum to 100 because of rounding.

Supplementary Table 6. Comparing pre-diagnosis and post-diagnosis total grey matter volume in each neurological condition using a general linear model controlling for other baseline characteristics

	Epilepsy			Stroke			Parkinson's disease			Migraine			Multiple Sclerosis			Motor Neurone disease		
	Full Model[a]			Full Model[a]			Full Model[a]			Full Model[a]			Full Model[a]			Full Model[a]		
	**β**	**t-stat**	**p**	**β**	**t-stat**	**p**	**β**	**t-stat**	**p**	**β**	**t-stat**	**p**	**β**	**t-stat**	**p**	**β**	**t-stat**	**p**
**Total Grey Matter volume**																		
Controls	Baseline			Baseline			Baseline			Baseline			Baseline			Baseline		
Pre-diagnosis	-0.05	-0.33	0.738	-0.23	-2.64	*0.008	-0.60	-3.23	*0.001	0.10	0.97	0.331	-0.64	-2.32	*0.020	-0.86	-3.10	*0.002
Post-diagnosis	-0.29	-4.71	*< .001	-0.34	-6.18	*< .001	-0.31	-2.13	0.033	0.05	1.89	0.059	-0.62	-6.84	*< .001	-0.26	-0.45	0.655

[a]*Full model: General linear model adjusted for baseline characteristics including age, sex, education, socioeconomic status and assessment centre.* β = *unstandardised betas*

Supplementary Table 7. Comparing pre-diagnosis and post-diagnosis total hippocampal volume in each neurological condition using a general linear model controlling for other baseline characteristics

	Epilepsy			Stroke			Parkinson's disease			Migraine			Multiple Sclerosis			Motor Neurone disease		
	Full Model[a]			Full Model[a]			Full Model[a]			Full Model[a]			Full Model[a]			Full Model[a]		
	**β**	**t-stat**	**p**	**β**	**t-stat**	**p**	**β**	**t-stat**	**p**	**β**	**t-stat**	**p**	**β**	**t-stat**	**p**	**β**	**t-stat**	**p**
**Total Hippocampal volume**																		
Controls	Baseline			Baseline			Baseline			Baseline			Baseline			Baseline		
Pre-diagnosis	-0.178	-1.11	0.26	-0.06	-0.69	0.49	-0.37	-2.00	0.05	-0.08	-0.80	0.43	-1.1	-3.99	*< .001	-0.74	-2.68	*0.01
Post-diagnosis	-0.264	-4.31	*< .001	-0.33	-6.03	*< .001	0.14	0.93	0.35	0.02	0.66	0.51	-1.17	-12.93	*< .001	0.34	0.60	0.55

[a]*Full model: General linear model adjusted for baseline characteristics including age, sex, education, socioeconomic status and assessment centre.* β = *unstandardised betas*

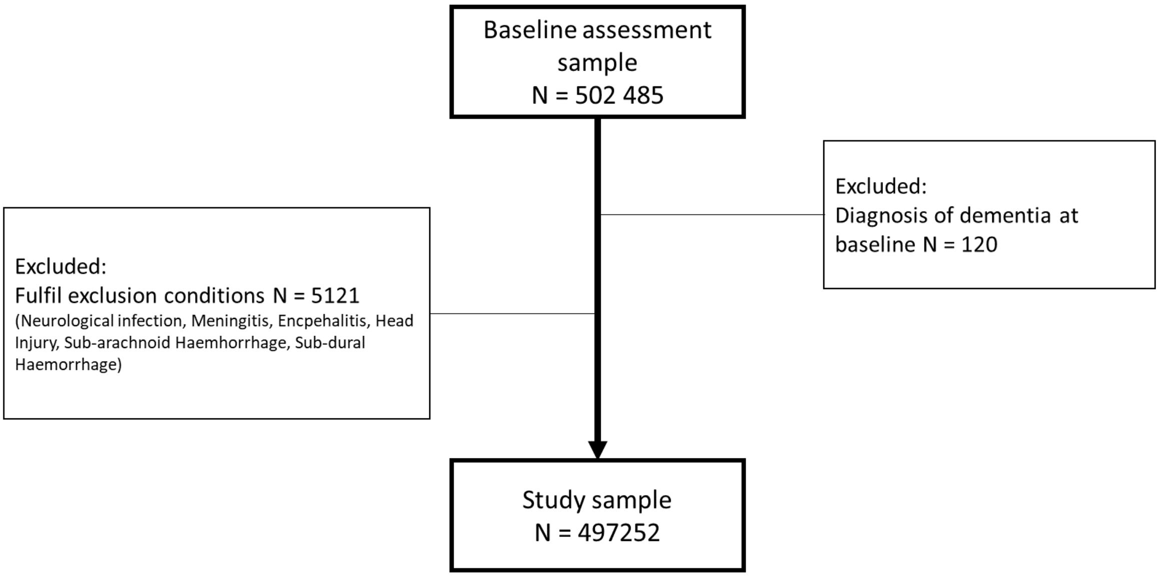

Supplementary Figure 1. Study flowchart

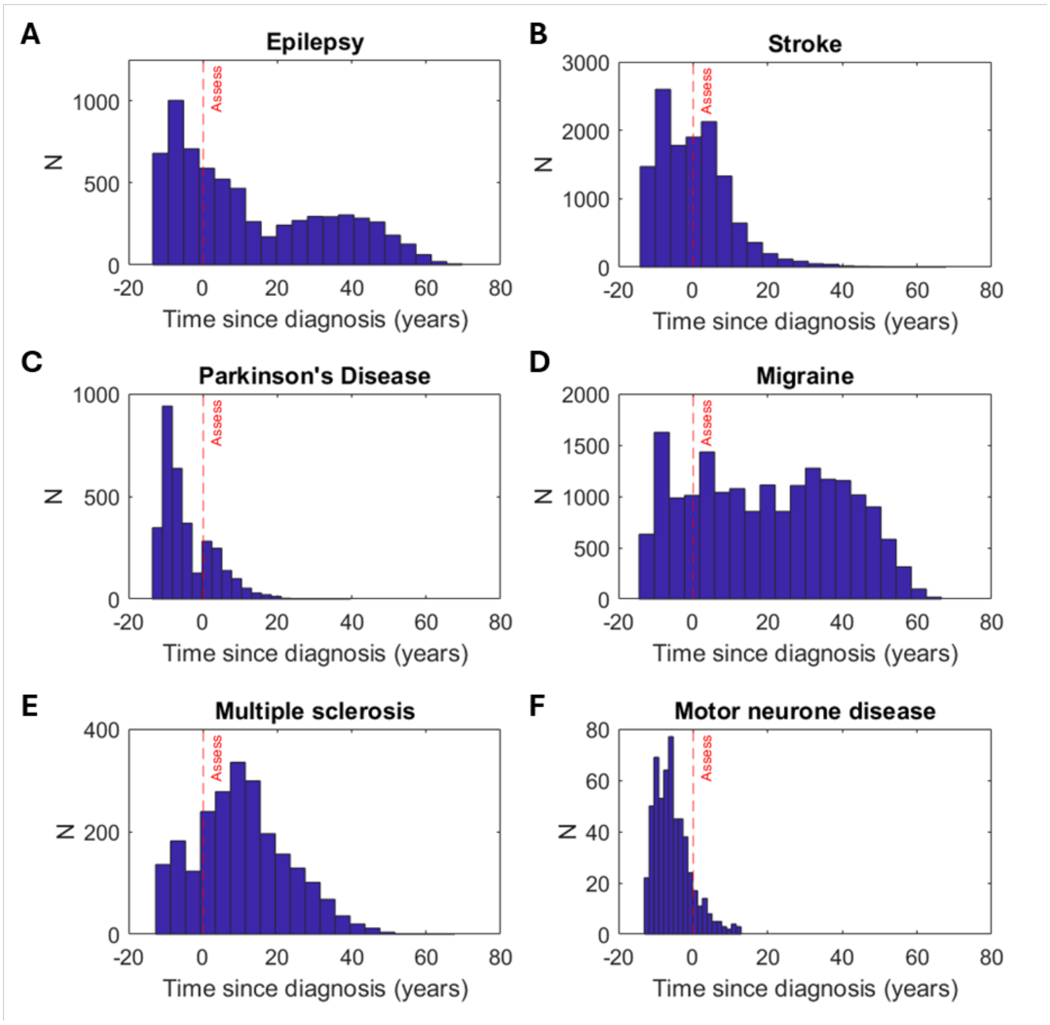

Supplementary Figure 2. Histogram of when neurological conditions were diagnosed in relation to study assessment.

Time-course of diagnoses across different neurological conditions. Red dotted line indicates the baseline study assessment. Negative time course values indicate that the participant will be diagnosed after initial assessment, during the follow-up period of the study.

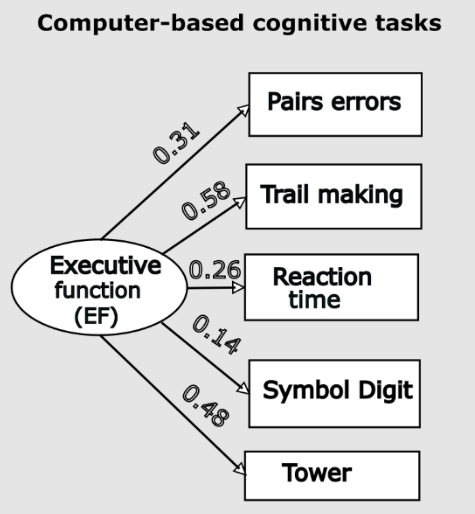

	Model fit
Comparative fit index (CFI)	0.990
Tucker-Lewis Index (TLI)	0.980
Root mean square error of approximation (RMSEA)	0.004
Standardised root mean square residual (SRMR)	0.017

Supplementary Figure 3. Confirmatory factor analysis of computer-based cognitive tasks

Path diagram (left) used to create a single latent variable of Executive Function (EF) using confirmatory factor analysis. Factor loadings shown on each arrow and fit indices shown on the right.

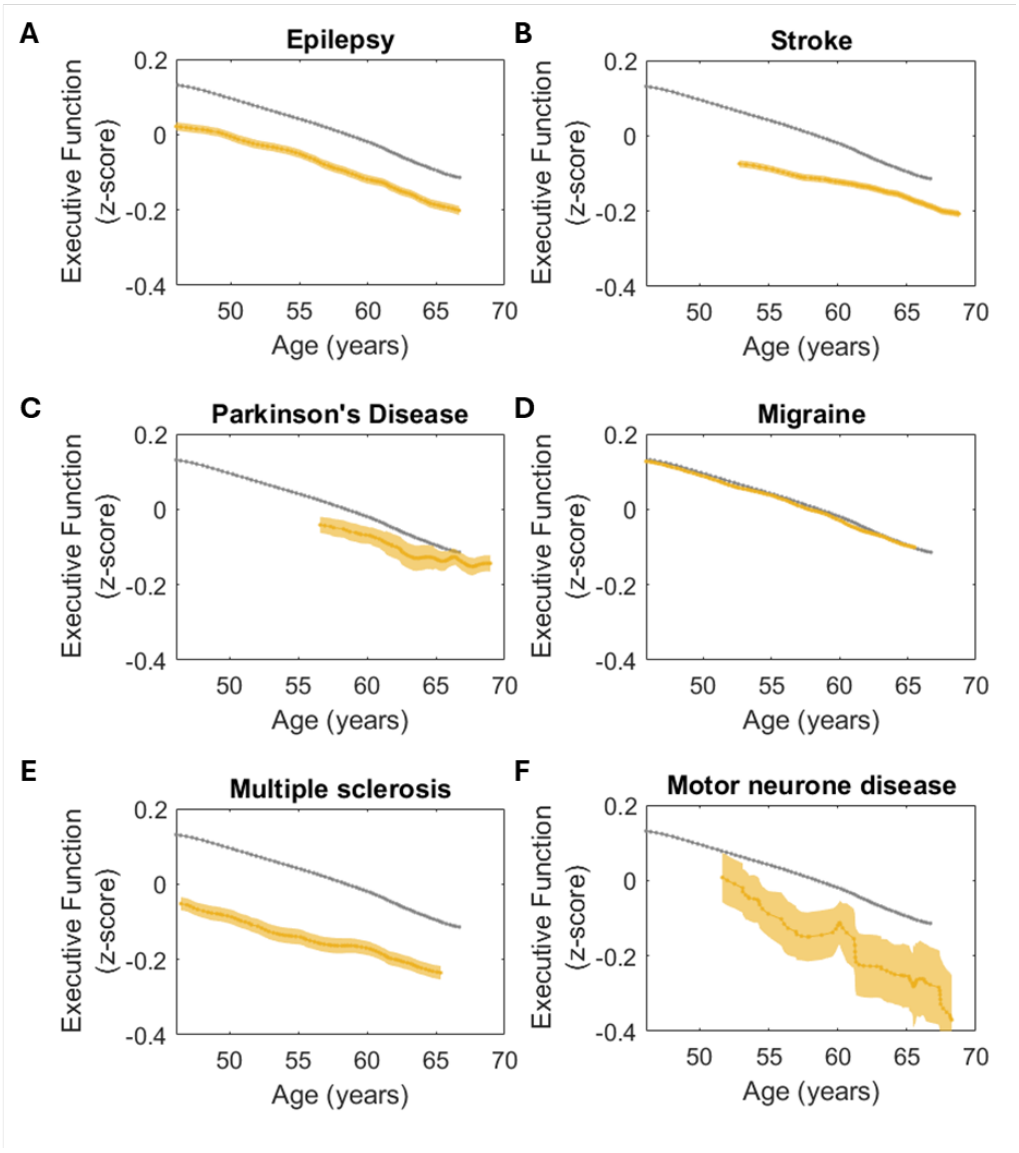

Supplementary Figure 4. Executive Function across age for different neurological conditions

Mean Executive Function declines across age for each neurological condition (yellow) compared with healthy controls (grey). Individuals with **A** epilepsy (N= 4247), **B** stroke (N=6352), **C** Parkinson's disease (N= 873), **E** multiple sclerosis (N= 1861) and **F** motor neurone disease (N=70) have lower Executive Function for a given age. **D** Migraine (N=14588) had a similar cognitive profile across age to controls. Error bars denote standard error.

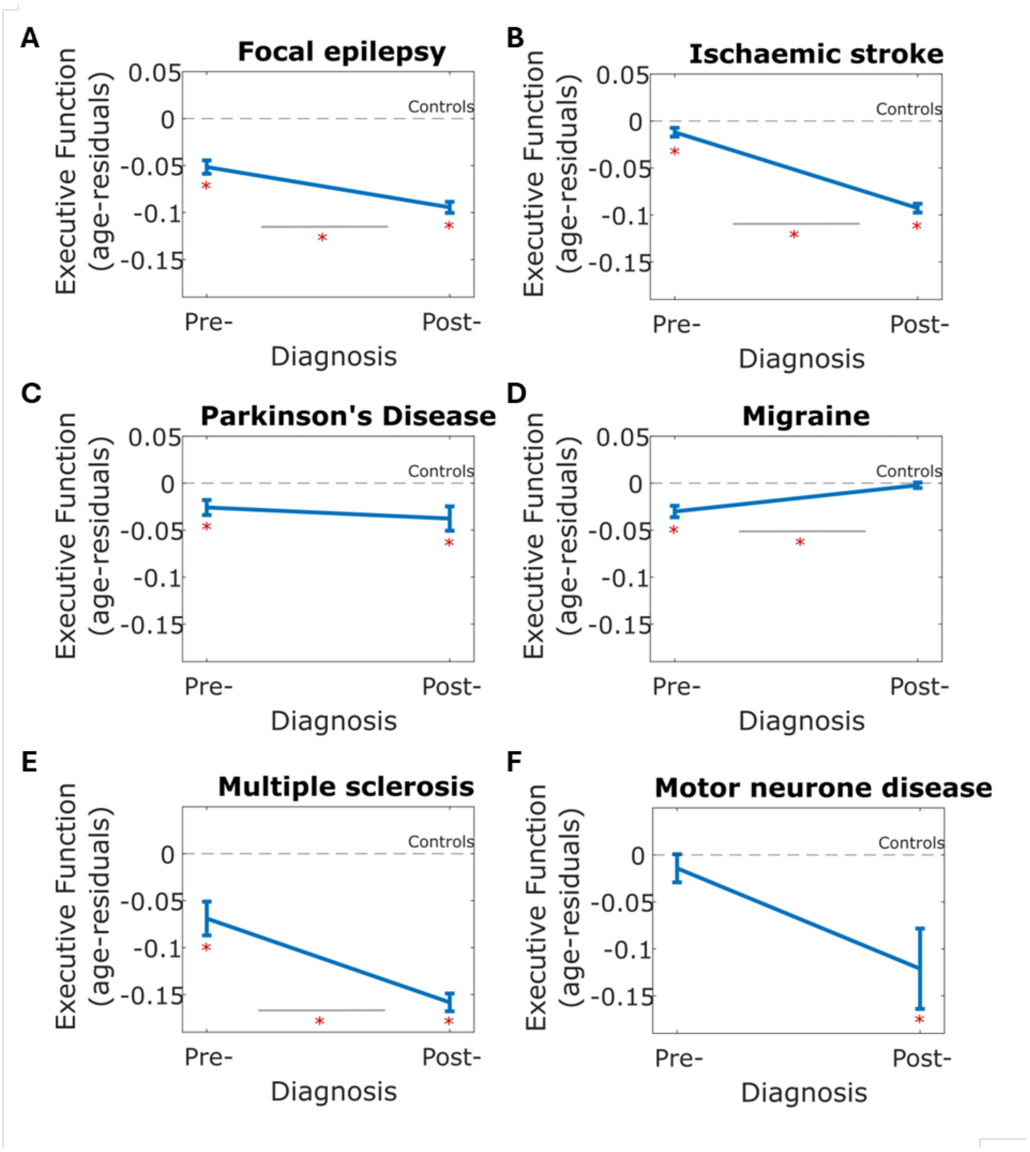

Supplementary Figure 5. Comparing pre-diagnosis and post-diagnosis Executive Function for each individual neurological condition

Executive Function was lower in post-diagnosis participants in all neurological conditions compared to pre-diagnosis participants apart from migraine and motor neurone disease. The level of pre-diagnosis Executive Function was lower than controls in all conditions (**A-E**) apart from **F** motor neurone disease. Mean executive function for each condition was compared with control group, asterix (*) below each error bar denotes significant difference p<0.05, while within-condition pre-diagnosis vs. post-diagnosis executive function was compared (represented by an * below grey bars) using a post-hoc Tukey analysis to account for multiple comparison. Individual group ANOVA statistics for focal epilepsy (N = 6758), ischaemic stroke (N = 12755), Parkinson's disease (N = 3315), migraine (N = 18254), multiple sclerosis (N = 2315), and motor neurone disease (N = 559) include: F = 189.70, $p < 0.001$; F = 235.08, $p < 0.001$; F = 14.31, $p < 0.001$; F = 18.48, $p < 0.001$; F = 202.40, $p <$ 0.001; and F = 5.05, $p < 0.001$, respectively. Post-hoc tukey values are found in Supplementary Table 3. Error bars denote standard error.

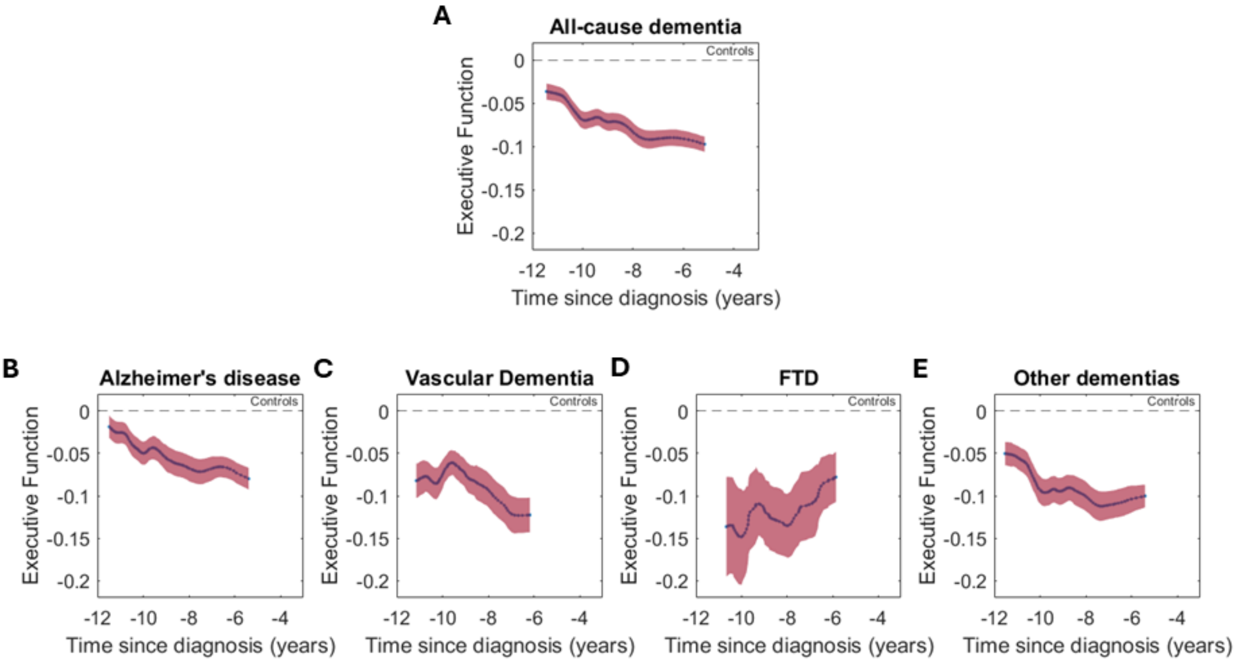

Supplementary Figure 6. Pre-diagnosis cognitive profile for participants with dementia

Executive Function declines progressively leading up to diagnosis of all-cause dementia as well as subtypes of Alzheimer's disease (N=2420), vascular dementia (N=1019) and other dementias (N=2325). Fronto-temporal dementia (N=199) does not show this change. *FTD – frontotemporal dementia*
